# Editorial: Promoting patient-centered care for pediatric rheumatology across Africa

**DOI:** 10.3389/fped.2025.1613257

**Published:** 2025-04-30

**Authors:** Wafa Hamdi, Angela Migowa

**Affiliations:** ^1^ Department of Rheumatology UR17SP04, Kassab Institute, Faculty of Medicine of Tunis, Tunis El Manar University, Tunis, Tunisia; ^2^ Department of Paediatrics and Child Health, Aga Khan University Medical College East Africa, Nairobi, Kenya

**Keywords:** pediatric rheumatology, patient-centred care, Africa, treatment access, juvenile idiopathic arthritis

**Editorial on the Research Topic**
Promoting patient-centered care for pediatric rheumatology across Africa

## Introduction

Pediatric rheumatology remains an evolving field in Africa, where challenges such as limited specialist availability, diagnostic delays, and inadequate public awareness hinder optimal patient care (1). Similarly, research in this field remains scarce, with a significant lack of data needed to define the spectrum and clinical expression of these diseases. Capturing real-world experiences and field reports is invaluable, as it provides critical insights to identify key areas for action and develop strategies to advance research in Africa.

This collection of six manuscripts offers valuable perspectives on various aspects of pediatric rheumatic diseases, including caregiver experiences, diagnostic challenges, therapeutic patient education, and regional advancements in the field. Together, these studies contribute to a more comprehensive understanding of the pediatric rheumatology landscape in Africa and underscore opportunities for progress.

## Empowering youth through therapeutic patient education (TPE) as a key driver for improved pediatric rheumatology care in Africa

In fact, empowering children with rheumatic diseases through TPE could be a transformative step toward better healthcare outcomes in Africa. Traditional patient education methods often fall short in equipping children with chronic conditions like Juvenile Idiopathic Arthritis (JIA) with essential self-management skills (2). Recognizing this gap, the *Pediatric Society of the African League Against Rheumatism* (PAFLAR) launched a TPE initiative, marking a significant advancement in patient-centered care. Through a structured TPE masterclass program, PAFLAR has trained healthcare providers and facilitated the implementation of TPE workshops in Kenya, Tunisia, and Nigeria. Initial findings indicate enhanced knowledge retention and self-management abilities among patients and their families. The success of this initiative underscores the potential of TPE in improving long-term disease outcomes and patient empowerment. Expanding this program across the continent could be a crucial step toward more comprehensive, patient-centered care in pediatric rheumatology.

## Understanding the caregiver experience in JIA as a key step toward better patient-centered care

Indeed the burden of JIA extends beyond the affected child, profoundly impacting caregivers and their families (1, 3, 4). This collection presents a qualitative study from Kenya that explores the experiences of parents caring for children with JIA at a tertiary referral hospital. Through in-depth interviews, the study identifies eight major challenges: medical-related difficulties, emotional distress, coping mechanisms, financial constraints, social challenges, interactions with healthcare personnel, and work-life disruptions. The findings highlight the critical need for a holistic, patient-centered approach that not only addresses the medical needs of children with JIA but also provides structured support for caregivers. Enhancing access to resources, developing targeted interventions, and increasing awareness among healthcare providers about the multidimensional impact of JIA are essential steps toward improving outcomes for both patients and their families.

## Challenges in diagnosing juvenile-onset systemic lupus erythematosus (JSLE) as a learning experience to improve healthcare

Thereby, the realities of clinical practice can sometimes be difficult to confront, particularly when it comes to delayed diagnoses in pediatric rheumatology. A striking case report from Bouaké, Ivory Coast, highlights the diagnostic challenges of JSLE, illustrating a 12-year journey before an accurate diagnosis was established. Initially misdiagnosed as malaria, sickle cell disease, and tuberculosis, the patient endured years of untreated disease activity, leading to severe complications. The diagnosis at the age of 17, underscores the urgent need for increased clinical suspicion, enhanced physician training, and improved diagnostic infrastructure in African healthcare settings. This case serves as a powerful reminder that early recognition and timely intervention are critical in preventing disease progression and reducing morbidity. Addressing these challenges can pave the way for better pediatric rheumatology care and improved patient outcomes across the continent.

## The evolution of pediatric rheumatology in Nigeria as an example of an implementation pathway for pediatric rheumatology

In fact, Nigeria's pediatric rheumatology landscape has witnessed gradual progress, yet significant gaps remain. This review provides a comprehensive look at the field's development, challenges, and future direction. While Lagos has made strides with the establishment of a dedicated pediatric rheumatology unit, specialized services remain scarce across most Nigerian states. Key obstacles identified in this review include low disease awareness, high diagnostic costs, a shortage of trained specialists, and limited research data. Addressing these challenges requires strategic investment in pediatric rheumatology training programs, expansion of specialized care units, and nationwide awareness campaigns.

## Juvenile-onset back pain in Cameroon: a decade of epidemiological insights as preliminary essential data with clinical implications

Thus, back pain in children and adolescents is often underestimated, yet it can serve as an early indicator of underlying rheumatic conditions (5). A retrospective study conducted in Douala, Cameroon, provides a decade-long overview of juvenile-onset back pain (JOBP), analysing clinical presentation and imaging findings. Among the 67 children diagnosed with JOBP, chronic mechanical lumbar pain was the most prevalent, related with imaging abnormalities such as disc disease and scoliosis. This study offers valuable long-term epidemiological data grounded in everyday clinical experience, reinforcing the need for early musculoskeletal assessment in pediatric patients with persistent back pain. These insights are crucial for shaping diagnostic strategies, improving clinical management, and guiding future research in pediatric rheumatology.

## Reliability of hip scoring systems in JIA as an insights from African researchers’ experience

In fact, accurate assessment tools are crucial for monitoring disease progression in JIA, especially in cases involving the hip joint. A Tunisian study in our collection examined the applicability and reliability of two hip scoring systems commonly used in pediatric rheumatology practice (6, 7). The results showed moderate to good reliability in evaluating joint space narrowing, erosion, and growth abnormalities. However, certain parameters, such as subchondral cysts and sclerosis, revealed poor concordance among observers. Significantly, the study found that targeted training led to improved agreement in some areas, underscoring the need for standardized training for pediatric rheumatologists before these tools can be broadly implemented in clinical practice. This research emphasizes the importance of collaborative studies and standardized methodologies in strengthening the pediatric rheumatology research network.

## Conclusion

This collection of studies highlights the progress, challenges, and opportunities in pediatric rheumatology across Africa. From caregiver burdens and diagnostic hurdles to the implementation of innovative Therapeutic Patient Education (TPE) and educational programs, each contribution deepens our understanding of the field. Looking ahead, enhanced training, increased research collaboration, and patient-centered strategies will be essential for improving outcomes for children with rheumatic diseases across the continent. This collection serves as a call to action for healthcare providers, policymakers, and researchers to address existing gaps and drive meaningful change in pediatric rheumatologic care, moving towards a more effective and patient-centred approach.
